# Oxygen Tolerance Domestication of *Blautia* sp. AUH-JLD56 Enables Efficient Aerobic Bioconversion of Arctigenin to 3′-Demethylarctigenin

**DOI:** 10.3390/microorganisms14071525

**Published:** 2026-07-13

**Authors:** Wenya You, Mingyue Liu, Hongkuan Ji, Zixuan Zhao, Hao Li, Xiuling Wang

**Affiliations:** College of Life Sciences, Hebei Agricultural University, Baoding 071001, China; youwya2023@163.com (W.Y.); lmyhbnd@126.com (M.L.); jhk10282026@163.com (H.J.); zzx02092026@163.com (Z.Z.)

**Keywords:** oxygen tolerance domestication, microbial bioconversion, arctigenin, 3′-demethylarctigenin (3′-DMAG)

## Abstract

Anaerobic bacteria are the dominant group in the animal intestinal microbiota, and most strains cannot grow normally upon exposure to air. *Blautia* sp. AUH-JLD56 (KF374935) is a strictly anaerobic strain previously isolated by our research group from human feces. Under anaerobic conditions, this strain converts arctigenin to 3′-demethylarctigenin (3′-DMAG), reaching a maximum conversion concentration of 3.6 mM. To improve the oxygen tolerance of this wild-type strain, we performed long-term oxygen tolerance domestication and successfully obtained an oxygen-tolerant mutant. Phenotypic analysis showed that the growth of the oxygen-tolerant mutant under aerobic conditions (OD_600nm_ = 2.37) was slightly lower than that of the wild-type under strictly anaerobic conditions (OD_600nm_ = 2.82). Compared with the wild-type, the mutant exhibited an accelerated aerobic growth rate and enabled the stable conversion of arctigenin. Notably, under aerobic conditions, the mutant achieved a maximum conversion concentration of 8.2 mM, which was significantly higher than the 3.6 mM obtained with the wild-type under anaerobic conditions. Under the respective optimal conditions, the average conversion rate and product yield for the domesticated strain (aerobic) in this study were 90.01% and 77.15%, while those for the non-domesticated strain (anaerobic) were 90.09% and 83.31%, respectively. This study realizes, for the first time, the efficient aerobic bioconversion of arctigenin to 3′-DMAG using an oxygen-tolerant derivative of a strict anaerobe. Our approach provides a new strategy and technical reference for the oxygen tolerance domestication and industrial application of other intestinal strict anaerobes.

## 1. Introduction

Microbial bioconversion technology utilizes one or more functional enzymes synthesized by microorganisms to catalyze the transformation of substrates at specific sites. It has already achieved industrial application in areas such as steroid hormone synthesis [1,2,3,4]. In recent years, this technology has been increasingly applied in the research and development of traditional Chinese medicine. *Arctii Fructus*, the dried ripe fruit of *Arctium lappa* L., is a commonly used traditional Chinese medicine known for its pharmacological effects, including clearing heat, reducing swelling, detoxifying, promoting eruption, and relieving sore throat. Arctiin and arctigenin are the most abundant lignan active components in *Arctii Fructus*. Existing studies have confirmed that arctigenin possesses various pharmacological activities, including anti-inflammatory [5], antitumor [6], renoprotective [7], antifibrotic [8], and neuroprotective [9] effects.

In clinical practice, traditional Chinese medicines are mostly administered orally. After oral administration, arctiin and arctigenin undergo metabolic transformation mediated by the gut microbiota. In 1992, Nose et al. [10] first confirmed that culturing arctiin with mouse fecal microbiota generated arctigenin and 3′-demethylarctigenin (3′-DMAG). In 2003, Xie et al. [11] identified six metabolites, including arctigenin, 3′-DMAG, and enterolactone, in an in vitro incubation system of arctiin with gut microbiota. In 2007, Jin et al. [12] isolated the first pure culture strain capable of transforming arctiin from human feces, *Eubacterium* sp. ARC-2, which metabolically converted arctiin into seven derivatives, including 3′-DMAG. In 2013, our research group isolated a strictly anaerobic bacterium, *Blautia* sp. AUH-JLD56, from human feces. Under anaerobic conditions, this strain catalyzes the directed conversion of arctiin or arctigenin into 3′-DMAG. When arctigenin was used as the substrate, the maximum conversion concentration reached 3.6 mM, with an average conversion rate as high as 90.5% [13].

The human gut microbiota plays a key role in maintaining host health, and related research has received widespread attention. Developing next-generation probiotics has become a promising technological strategy for regulating the gut microbiota and improving host health [14]. Strictly anaerobic bacteria account for 90~99% of the gut microbiota and represent the core group of the gut microbial community [15,16]. However, due to limitations in anaerobic culture conditions and the fact that most strictly anaerobic bacteria rely on synergistic interactions between strains for optimal growth, the availability of gut-specific strains that can be successfully isolated, identified, and functionally characterized remains very scarce. At the same time, the natural source of 3′-DMAG is rare and difficult to obtain, leading to a lack of systematic studies on its biological activities. Previous research by our group confirmed that at the same concentration, 3′-DMAG exhibits significantly better DPPH radical scavenging activity than its precursor compound, arctigenin [13]. In vivo animal experiments further demonstrated that 3′-DMAG significantly inhibits tumor growth without obvious toxic side effects [6]. Given that the microbial bioconversion products of arctiin and arctigenin possess superior biological activities, this research direction has become a hot topic in the field. However, strictly anaerobic bacteria are highly sensitive to oxygen and cannot grow normally under conventional aerobic conditions. Their culture and bioconversion processes both rely on a strictly anaerobic environment, and anaerobic systems have drawbacks such as high maintenance costs and large equipment investments, which severely limit the functional mechanistic analysis and industrial transformation application of these strains [17]. Therefore, establishing effective strategies to enhance the oxygen tolerance of strictly anaerobic bacteria and achieve their stable growth under aerobic conditions is a key direction for breaking through current research bottlenecks.

Currently, research on improving oxygen tolerance in strictly anaerobic bacteria remains relatively limited. Clostridia are typical obligate anaerobes, most of which are highly sensitive to oxygen, and existing studies have begun to explore their regulatory mechanisms of oxygen tolerance. In 2007, Riebe et al. [18] demonstrated that the desulfoferrodoxin produced by the obligate anaerobe *Clostridium acetobutylicum* exhibits superoxide reductase activity and is a core functional component for intracellular reactive oxygen species scavenging. In 2008, Hillmann et al. [19] knocked out the homologue of the peroxide repressor protein in *C. acetobutylicum*, and the resulting mutant showed a significantly prolonged oxygen tolerance time and could achieve limited growth under aerobic conditions. In 2014, Zhang et al. [20] performed a comparative genomic reconstruction analysis of the Rex regulator in 11 *Clostridium* species, revealing that Rex responds to changes in the intracellular NADH/NAD^+^ ratio and regulates downstream gene expression, thereby mediating fermentation product synthesis and oxidative stress tolerance in *C. acetobutylicum*. The strictly anaerobic bacterium *Faecalibacterium prausnitzii* is a core commensal species with a high abundance and high detection rate in the human gut and is closely associated with host health. In 2023, Khan et al., while retaining its core probiotic functions, adaptively modified *F. prausnitzii* and successfully endowed the strain with oxygen tolerance; the modified strain could grow syntrophically with the sulfate-reducing bacterium *Desulfovibrio piger* and produce butyrate, providing an important reference for the development of next-generation strictly anaerobic probiotics [21]. Furthermore, previously in 2015, our research group, by gradually reducing the concentration of reducing agents in the culture medium and decreasing the depth of liquid medium, domesticated the strictly anaerobic bacterium *Clostridium* sp. AUH-JLC108 isolated from cock feces into an oxygen-tolerant mutant strain *Clostridium* sp. Aeroto-AUH-JLC108, thereby achieving the efficient synthesis of *O*-desmethylangolensin from daidzein under aerobic conditions [22].

In this study, the strictly anaerobic bacterium *Blautia* sp. AUH-JLD56 [13], previously isolated by our research group, was used as the starting strain. Through continuous oxygen tolerance domestication, a stable oxygen-tolerant mutant strain was obtained. This mutant strain can efficiently catalyze the conversion of arctigenin into the active product 3′-DMAG in the presence of atmospheric oxygen, achieving a maximum conversion concentration of 8.2 mM. This study accomplished, for the first time, the efficient aerobic biosynthesis of 3′-DMAG, overcoming the dependence of the transformation process of this type of strain on a strictly anaerobic environment, and also provides new strategies and insights for the oxygen tolerance domestication and industrial application of gut strictly anaerobic bacteria.

## 2. Materials and Methods

### 2.1. Culture Media and Chemicals

Brain heart infusion medium (BHI, Becton, Dickinson and Company, Franklin Lakes, NJ, USA) was used to cultivate strictly anaerobic intestinal bacteria.

Adaptation medium: based on BHI medium, supplemented with 0.08% agar, 0.15% L-cysteine, and 0.15% L-ascorbic acid. Acclimatization medium: based on the activation medium, the amounts of agar and reducing agents were gradually reduced until the final concentrations of agar, L-cysteine, and ascorbic acid all reached zero. Agar, L-cysteine, and ascorbic acid were from Beijing Solarbio Science & Technology Co., Ltd., Beijing, China. Arctigenin standard (purity > 98%) was purchased from Shanghai Yuanye Bio-Technology Co., Ltd., Shanghai, China. The 3′-demethylarctigenin standard was prepared using our previously described microbial biotransformation method [13].

### 2.2. Test Strain

*Blautia* sp. AUH-JLD56 is a strictly anaerobic bacterium previously isolated by our research group from human fecal samples [13]. The master stock was preserved in a 10% skim milk solution (Inner Mongolia Yili Industrial Group Co., Ltd., Hohhot, China), overlaid with a 2 mm thick layer of liquid paraffin (Tianjin Damao Chemical Reagent Factory, Tianjin, China), and stored at −80 °C.

### 2.3. Oxygen-Tolerance Domestication Process

The strictly anaerobic bacterium *Blautia* sp. AUH-JLD56 preserved in an ultra-low temperature freezer was inoculated into 4 mL of freshly sterilized BHI medium at an inoculum size of 10% (*v*/*v*). The total cell count of the bacterial suspension was determined using a hemocytometer, and the initial inoculum density was 2.14 × 10^9^ cells/mL. The culture was incubated in an anaerobic chamber (BAKER Ruskinn, Ruskinn Ltd., Bridgend, UK) under an atmosphere of CO_2_:H_2_:N_2_ (5:10:85) at 37 °C for 24 h under static conditions. The pre-cultured broth of the strictly anaerobic strain AUH-JLD56 was then inoculated at an inoculum volume of 10% (*v*/*v*) into the above-mentioned adaptation medium and incubated at 37 °C in a biochemical incubator (ZXDP-A2160, Shanghai Zhicheng Analytical Instrument Manufacturing Co., Ltd., Shanghai, China) for 24 h. This culture process was repeated five times, and the entire procedure was designated as the “first round of acclimatization”. After the first round of acclimatization, the substrate arctigenin was added to the culture broth, and the mixture was cultured for 3 days. The culture was then extracted with ethyl acetate, evaporated to dryness, redissolved in 100% methanol, and the bioconversion of the substrate arctigenin was analyzed by high-performance liquid chromatography (HPLC). The specific HPLC method is detailed in the section on HPLC detection, preparation, and the identification of arctigenin transformation products.

The specific domestication procedures were carried out as follows. Ascorbic acid and L-cysteine were used as reducing agents to lower the redox potential of the medium. L-cysteine and agar were added to the liquid BHI medium prior to sterilization to final concentrations of 0.15% and 0.08%, respectively. After sterilization, ascorbic acid was filter-sterilized through a 0.22 μm membrane and supplemented to a final concentration of 0.15%. The BHI medium containing these three supplements was designated as the initial medium. After five serial subcultures of the anaerobic strain in this initial medium, the OD_600nm_ and the bioconversion capacity toward the substrate arctigenin were determined under aerobic conditions. If neither growth nor bioconversion activity declined, the ascorbic acid concentration was decreased stepwise by 0.01% per passage until it reached 0%. Following the same protocol, the L-cysteine concentration was gradually reduced from 0.15% to 0%, and finally, the agar concentration was stepwise decreased from 0.08% to 0%. The resulting oxygen-tolerant mutant was designated as the aerotolerant strain Aeroto-AUH-df6. The detailed domestication procedure for oxygen tolerance is presented in Table 1. It should be noted that if the strain’s biomass accumulation or bioconversion capacity deteriorated during domestication, the reduction gradient of 0.01% per passage was adjusted to 0.005%.

### 2.4. Cell Morphology, Growth Property, Physiological and Biochemical Indicators

The strictly anaerobic bacterium *Blautia* sp. AUH-JLD56 and the oxygen-tolerant strain Aeroto-AUH-df6 were each cultured to the mid-to-late exponential growth phase. The cell morphology of the strains was observed under a light microscope (CN15-T31, KONKYO, Beijing, China) at 40× magnification. During the same period, the OD_600nm_ and pH of the culture broth were measured at fixed time intervals, and the growth curves as well as the change in pH over time were plotted. Biochemical indicator changes before and after oxygen-tolerant domestication were determined using the API20 Kit (bioMérieux, Lyon, France). Biochemical tests not included in the kit were performed according to the procedures described in the Wadsworth-KTL Anaerobic Bacteriology Manual [23].

### 2.5. Determination of the 16S rRNA Gene Sequence of the Oxygen-Tolerant Strain

Genomic DNA was extracted from the strain with minor modifications to the protocol described by Minas et al. [24]. Briefly, 1.0 mL of bacterial culture at the mid-to-late exponential phase was centrifuged at 15,300× *g* for 10 min, and the supernatant was discarded. The pellet was washed once with 400 μL of sterile water, followed by another round of centrifugation to remove the supernatant. Then, 20 μL of lysozyme solution (50 mg/mL) was added to the pellet, and the mixture was incubated overnight at 37 °C. Subsequently, 400 μL of CTAB lysis buffer (containing 12.11 g/L Tris, 81.82 g/L NaCl, 20 g/L CTAB, and 7.4448 g/L EDTA) was added, and the sample was incubated in a 65 °C water bath for 30 min. An equal volume of phenol–chloroform mixture (1:1, *v*/*v*) was added, and the solution was mixed thoroughly for 5 min. After centrifugation at 15,300× *g* for 10 min, the aqueous supernatant was retained, and this phenol–chloroform extraction was performed once more. Two volumes of absolute ethanol were then added and mixed well, and the mixture was allowed to stand for 10 min. Following centrifugation at 15,300× *g* for 5 min, the supernatant was discarded, and the pellet was washed with 1 mL of 70% aqueous ethanol to obtain the genomic DNA. Finally, the DNA pellet was fully dissolved in 20 μL of sterile water for subsequent use.

The 16S rRNA gene was amplified by PCR using the universal primers 27F (5′-AGAGTTTGATCCTGGCTCAG-3′) and 1492R (5′-GGTTACCTTGTTACGACTT-3′), and the amplicons were subsequently sent to Bioengineering (Shanghai) Co., Ltd. (Shanghai, China) for DNA sequencing. The PCR reaction mixture (total volume 40 μL) comprised 20 μL of 2× San Taq Fast PCR Master Mix, 2 μL of each primer, 2 μL of DNA template, and 14 μL of sterile water. The thermal cycling program was performed on a PCR Amplifier (2720, Applied Biosystems, Waltham, MA, USA) and set as follows: initial denaturation at 94 °C for 5 min; 29 cycles of denaturation at 94 °C for 1 min, annealing at 55 °C for 30 s, and extension at 72 °C for 90 s; followed by a final extension at 72 °C for 10 min. The 16S rRNA sequence was aligned with that of the unacclimated strain, and a phylogenetic tree was constructed based on this alignment.

### 2.6. Determination of Bacterial Cell Counts and Cell Dry Weight

Bacterial cells were enumerated by gradient dilution, while dry weight was measured by the centrifugation-drying gravimetric method. One milliliter of the bacterial suspension was transferred into a pre-weighed 2 mL centrifuge tube that had been dried to constant weight (recorded as *m*_1_). The sample was centrifuged at 2700× *g* for 10 min, and the supernatant was discarded. The pellet was resuspended in 1 mL of sterile water by vortexing, followed by centrifugation under the same conditions and removal of the supernatant. This washing step was repeated three times. The washed pellet, along with the tube, was placed in a forced-air drying oven at 105 °C and dried to constant weight. After cooling to room temperature in a desiccator, the tube was accurately weighed, and the weight was recorded as *m*_2_. All experiments were performed in triplicate. The bacterial dry weight (*R*) was calculated using the following formula:

(1) $$R=\frac{{m_{2}-m}_{1}}{V}$$

where *R*: the bacterial dry weight (mg/mL); *m*_1_: the weight of the empty tube (mg); *m*_2_: the weight of the tube containing the dried pellet (mg); *V*: the volume of the bacterial suspension (mL).

### 2.7. Determination of Oxygen Tolerance of the Oxygen-Tolerant Strain

To investigate the influence of the medium height on bacterial growth, the oxygen-tolerant strain Aeroto-AUH-df6 was inoculated at a 10% inoculum amount into fresh BHI liquid medium with different liquid depths (i.e., liquid medium heights of 2, 1, and 0.75 cm, respectively). Under the same subculture and culture conditions, each setup was subcultured five times. Similarly, to study the effect of inoculum size on the growth of the oxygen-tolerant strain Aeroto-AUH-df6, the strain was inoculated at different inoculum amounts (specifically 10%, 5%, 2.5%, and 1.25%) into 4 mL of freshly sterilized BHI medium. Under the same subculture and culture conditions, each setup was subcultured five times. The OD value of the oxygen-tolerant strain Aeroto-AUH-df6 was measured at a UV absorption wavelength of 600 nm. The experiment was repeated three times.

### 2.8. HPLC Detection and Structural Identification of Arctigenin Bioconversion Products

After culturing the oxygen-tolerant strain Aeroto-AUH-df6 with the substrate arctigenin for 3 days, 1 mL of the culture broth was taken and extracted with an equal volume of ethyl acetate. The extract was evaporated to dryness, reconstituted in 100% methanol, filtered through an organic membrane, and then analyzed by HPLC (Waters 2695 system equipped with a photodiode array detector, Waters Corporation, Milford, MA, USA). The chromatographic column was a Kromasil C_18_ column (250 mm × 4.6 mm, 5 μm). Mobile phase A was 10% acetonitrile in water containing 0.1% acetic acid, and mobile phase B was 90% acetonitrile in water containing 0.1% acetic acid. The elution method was A:B = 60:40 (*v*/*v*) at a flow rate of 1 mL/min, injection volume 20 μL, and detection wavelength 280 nm. The semi-preparative chromatographic conditions for the product of arctigenin converted by the oxygen-tolerant strain Aeroto-AUH-df6 were as follows: a Kromasil C_18_ preparative column (250 mm × 10 mm, 5 μm), flow rate 2.0 mL/min, and detection wavelength 280 nm. The bioconversion products were analyzed by mass spectrometry (ESI-MS) on a Bruker Daltonics Apex-Ultra mass spectrometer (Billerica, MA, USA) and by ^1^H and ^13^C NMR spectroscopy in CDCl_3_ on a Bruker AVANCE NMR spectrometer (400 MHz, Billerica, MA, USA).

### 2.9. Determination of the Bioconversion Dynamics of Arctigenin by the Strain Before and After Domestication

A total of 10 mL of the strictly anaerobic bacterium *Blautia* sp. AUH-JLD56 culture and the oxygen-tolerant strain Aeroto-AUH-df6 culture were inoculated into 100 mL of fresh BHI liquid medium in an anaerobic chamber and a clean bench, respectively. The substrate arctigenin was added to a final concentration of 0.4 mM. Samples of the anaerobic strain were taken every 4 h within 24 h, while samples of the oxygen-tolerant strain were taken every 2 h for 24 h. Each sample was tested in triplicate. Substrate reduction and metabolite production were measured by HPLC. Curves showing changes in substrate and product concentrations over culture time were plotted for both the anaerobic and the oxygen-tolerant strain cultures.

### 2.10. Determination of the Transformation Capacity of Arctigenin by the Strain Before and After Domestication

The strictly anaerobic bacterium *Blautia* sp. AUH-JLD56 and the oxygen-tolerant strain Aeroto-AUH-df6 were inoculated at a 10% inoculum amount into 4 mL of fresh BHI liquid medium in an anaerobic chamber and a clean bench, respectively, and different concentrations of the substrate arctigenin were added. The final concentrations of arctigenin added to culture with the strictly anaerobic strain AUH-JLD56 in the anaerobic chamber were 0.6 mM, 1.2 mM, 2.4 mM, 3.6 mM, and 4.0 mM. The final concentrations of arctigenin added to the culture with the oxygen-tolerant strain Aeroto-AUH-df6 in a conventional biochemical incubator were 4.6 mM, 5.8 mM, 7.0 mM, 8.2 mM, and 9.4 mM. When the final concentration of arctigenin added exceeded 2.5 mM, the substrate was added in 2–4 portions to avoid the inhibition of bacterial growth caused by an excessively high single addition. The time interval between two consecutive substrate additions was 4 h. After 3 days of culture, the transformation of the substrate arctigenin was analyzed by HPLC. The experiment was repeated three times.

The calculation formulas for the substrate conversion rate and the average product formation rate are as follows:

(2) $$R_{1}=\frac{C_{1}}{C_{1}+C_{2}}\times 100\%$$

(3) $$R_{2}=\frac{C_{1}+C_{2}}{C}\times 100\%$$

where *R*_1_: conversion rate; *R*_2_: product yield; *C*_1_: product concentration (mM); *C*_2_: concentration of the unconverted residual substrate (mM); *C*: initial concentration of the substrate added to the medium (mM).

### 2.11. Growth and Bioconversion Capacity of the Oxygen-Tolerant Strain Under Anaerobic Conditions

The genetic stability of the aerotolerant mutant was preliminarily assessed by comparing its growth and bioconversion activity toward the substrate arctigenin under both aerobic and anaerobic conditions. The cryopreserved aerotolerant strain Aeroto-AUH-df6 was reactivated under normal aerobic conditions and consecutively subcultured for five passages in the presence of atmospheric oxygen until its growth stabilized in an aerobic environment. Following these five passages, the maximum OD_600nm_, pH, and arctigenin bioconversion efficiency of strain Aeroto-AUH-df6 were determined under aerobic conditions, with substrate concentrations adjusted to 5.8 mM and 8.2 mM, respectively. The procedures for substrate addition, extraction, and detection were the same as described above.

To evaluate whether anaerobic conditions affect the growth and bioconversion performance of the aerotolerant strain, the aerobically stabilized Aeroto-AUH-df6 was cultured in an anaerobic chamber (gas composition: CO_2_:H_2_:N_2_ = 5:10:85, 37 °C). The strain was inoculated into 4 mL of fresh BHI medium at a 10% (*v*/*v*) inoculum size and incubated statically, with subculturing performed every 12 h for five consecutive passages within the anaerobic chamber. The OD_600nm_ and pH values of the bacterial suspensions were measured prior to each passage. To investigate the impact of anaerobic conditions on the bioconversion capability of Aeroto-AUH-df6, the strain after five successive anaerobic passages was used as the seed culture. It was inoculated into 4 mL of fresh liquid BHI medium at a 10% inoculation ratio inside the anaerobic chamber, with arctigenin added to final concentrations of 5.8 mM and 8.2 mM, respectively. Following a 3-day anaerobic incubation, its substrate bioconversion capacity under anaerobic conditions was quantified.

### 2.12. Statistical Analysis

All data were statistically analyzed using IBM SPSS Statistics 26.

## 3. Results

### 3.1. Cell Morphology and Growth Properties of the Oxygen-Tolerant Domesticated Strain

**Figure 1:**
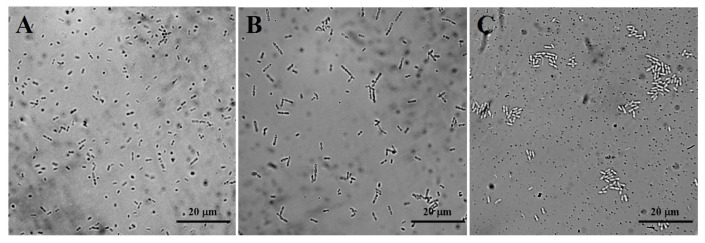
Light micrographs showing (**A**) elliptical cells of the original strictly anaerobic bacterial strain AUH-JLD56 grown anaerobically; (**B**) elongated cells during the oxygen tolerance domestication process; and (**C**) cells of the finally obtained oxygen-tolerant mutant strain Aeroto-AUH-df6 grown aerobically. The bacterial cells were sampled in the late exponential phase.

Through oxygen tolerance domestication, we successfully obtained an oxygen-tolerant mutant strain, designated as the oxygen-tolerant strain Aeroto-AUH-df6, from the strictly anaerobic bacterium *Blautia* sp. AUH-JLD56. This oxygen-tolerant strain exhibits significant differences in multiple biological characteristics compared with the wild-type strain *Blautia* sp. AUH-JLD56. Observations under an optical microscope revealed that the wild-type strictly anaerobic strain *Blautia* sp. AUH-JLD56, cultured in an anaerobic chamber, displays oval-shaped cells that mostly exist as single cells, with a few appearing as short rods formed by two connected cells (Figure 1A). During the oxygen-tolerance domestication process, cell length first increased (Figure 1B), then significantly decreased, eventually approaching that of the original strain. Ultimately, the obtained oxygen-tolerant strain Aeroto-AUH-df6, when cultured in a conventional biochemical incubator, appears as short rods forming chains of two connected cells (Figure 1C).

**Figure 2:**
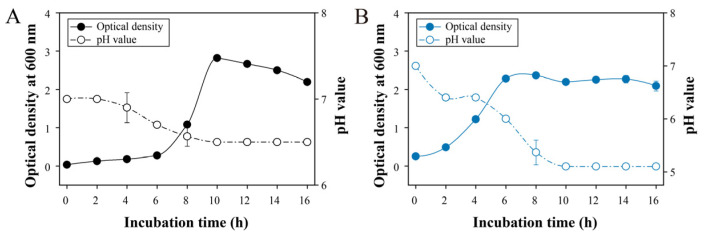
Time course of bacterial cell growth (solid line) and pH change (dashed line) for the original strictly anaerobic strain AUH-JLD56 grown anaerobically (**A**) and the oxygen-tolerant mutant strain Aeroto-AUH-df6 grown aerobically (**B**). The inoculum concentration was 10% (*v*/*v*). The culture broth of each strain was sampled every 2 h to measure both pH and OD_600nm_. Error bars represent the standard deviation of biological replicates.

Compared with the wild-type strain AUH-JLD56 cultured in the anaerobic chamber (Figure 2A, solid line), the oxygen-tolerant strain Aeroto-AUH-df6 cultured in the conventional biochemical incubator showed a significantly higher growth rate. However, the maximum OD_600nm_ of this strain averaged 2.37, which was lower than the average maximum OD_600nm_ of 2.82) observed for the wild-type strain AUH-JLD56 under anaerobic conditions. It should be noted that the cell dry weight of strain Aeroto-AUH-df6 cultured under aerobic conditions was 1.08 mg/mL, slightly higher than that of the strain cultivated under anaerobic conditions (1.03 mg/mL). In addition, the dynamic characteristics of the growth processes differed markedly: the OD_600nm_ of the wild-type strain AUH-JLD56 exhibited a continuous decline after 10 h of inoculation. In contrast, the oxygen-tolerant strain Aeroto-AUH-df6 showed a brief decrease in biomass between 8 and 10 h post-inoculation, followed by a rapid entry into the stationary phase, during which the OD_600nm_ did not show any obvious decline (Figure 2B, solid line). Regarding dynamic changes in the pH of the cultural broth, the pH of the wild-type strain AUH-JLD56 continuously decreased from 7.0 to approximately 6.5 within 0~10 h post-inoculation and then remained stable (Figure 2A, dashed line). The pH changes of the oxygen-tolerant strain Aeroto-AUH-df6 displayed multi-stage characteristics: a rapid decrease within 0~2 h post-inoculation, followed by a slight increase over the next 2 h, then a second rapid decrease, ultimately stabilizing at around 5.1 (Figure 2B, dashed line).

### 3.2. Variation of Physiological and Biochemical Indicators Before and After Domestication

Before domestication, the strictly anaerobic bacterium *Blautia* sp. AUH-JLD56 exhibited negative urease activity. After oxygen tolerant domestication, the resulting mutant strain Aeroto-AUH-df6 showed positive urease activity. Compared with the original strain *Blautia* sp. AUH-JLD56, the oxygen-tolerant strain Aeroto-AUH-df6 demonstrated significantly enhanced carbon source metabolic utilization capability. The original strain AUH-JLD56 was unable to utilize sucrose, xylose, raffinose, rhamnose, arabinose, mannose, trehalose, melezitose, cellobiose, glycerol, salicin, or sorbitol, whereas the oxygen-tolerant strain Aeroto-AUH-df6 acquired the ability to metabolize and utilize all of the above carbon sources (Table 2).

**Figure 3:**
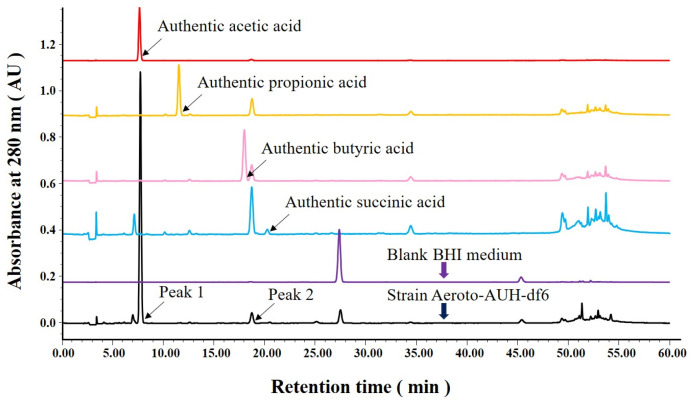
HPLC elution profile of short-chain fatty acids (SCFAs) produced in the culture medium of the oxygen-tolerant strain Aeroto-AUH-df6.

In previous studies, we found that the strictly anaerobic strain *Blautia* sp. AUH-JLD56 exhibits extremely weak acid-producing capacity. After anaerobic cultivation, the pH of its culture broth stabilized at approximately 6.5 from an initial value of 7.0. When the aerotolerant mutant Aeroto-AUH-df6 was cultured under aerobic conditions, the pH of its culture medium dropped from 7.0 to a steady level of around 5.1 (Figure 2). To identify the predominant acid species responsible for the pH reduction in the culture broth of Aeroto-AUH-df6, we quantified its short-chain fatty acid production. The results revealed that Aeroto-AUH-df6 accumulated substantial acetic acid during growth (Figure 3).

### 3.3. 16S rRNA Gene Sequence Analysis of the Oxygen-Tolerant Domesticated Strain

**Figure 4:**
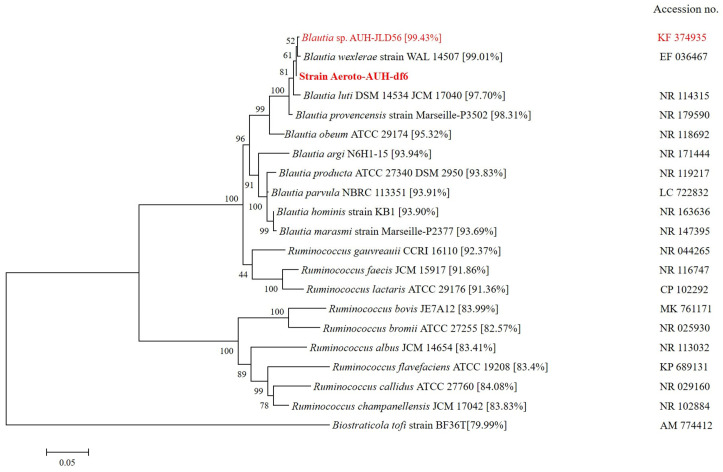
Phylogenetic tree showing the relationship of the oxygen-tolerant strain Aeroto-AUH-df6 to related taxa based on 16S rRNA gene sequences. Wherein* Blautia* sp. AUH-JLD56 for the non-domesticated wild-type strain, and strain Aeroto-AUH-df6 for the oxygen-tolerant mutant.

The 16S rRNA gene of the oxygen-tolerant strain Aeroto-AUH-df6 was sequenced, and a phylogenetic tree was constructed together with the original non-domesticated strain AUH-JLD56 (Figure 4). BLAST (https://blast.ncbi.nlm.nih.gov/Blast.cgi, 18 April 2024) sequence alignment showed that the oxygen-tolerant strain Aeroto-AUH-df6 shared 99.43% sequence similarity with the original strain *Blautia* sp. AUH-JLD56, and 99.01% similarity with *Blautia* *wexlerae * strain WAL 14507. Phylogenetic analysis indicated that this oxygen-tolerant strain clustered within the same evolutionary branch as the *Blautia* genus strains. In conclusion, the mutant strain obtained through oxygen-tolerant domestication was identified as belonging to the genus *Blautia* and named *Blautia* sp. Aeroto-AUH-df6.

Additionally, the 16S rRNA gene sequences of the oxygen-tolerant strain Aeroto-AUH-df6 and its parental wild-type strain *Blautia* sp. AUH-JLD56 (GenBank accession no. KF374935) were aligned against the wild-type reference sequence. Compared with the reference, three base insertions were identified in strain Aeroto-AUH-df6: an adenine (A) insertion between positions 12 and 13, a guanine (G) insertion between positions 1393 and 1394, and another G insertion between positions 1404 and 1405. In addition, three point mutations were detected: A → T at position 53, A → C at position 1063, and T → A at position 1126. All nucleotide positions are numbered according to the corresponding reference sequence deposited in NCBI.

### 3.4. Analysis of Oxygen Tolerance of the Oxygen-Tolerant Domesticated Strain

**Figure 5:**
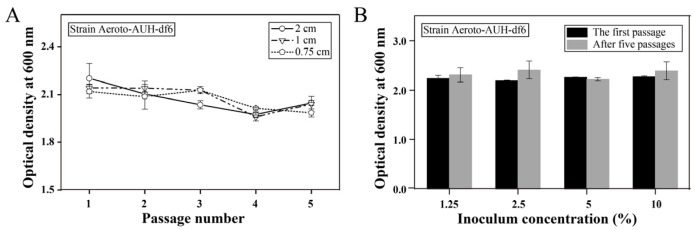
Influence of culture depth (**A**) and inoculum concentration (**B**) on cell growth of the oxygen-tolerant strain Aeroto-AUH-df6 in the presence of atmospheric oxygen. Cultures with a final OD_600nm_ of 2.0 were used as inoculum. Growth yield was determined every 8 h, followed by continuous passaging for five generations. Error bars represent the standard deviation of biological replicates.

Equal amounts of the oxygen-tolerant strain Aeroto-AUH-df6 were inoculated into liquid media with different liquid layer heights (2 cm, 1 cm, 0.75 cm). The results showed that the liquid layer height had no significant effect on the mean maximum OD_600nm_ of this oxygen-tolerant mutant strain (Figure 5A). Further investigation into the effect of different inoculum sizes on strain growth revealed that even when the inoculum amount was reduced to 1.25%, the oxygen-tolerant strain Aeroto-AUH-df6 could still proliferate stably after five consecutive passages (Figure 5B), indicating that reducing the inoculum size did not significantly inhibit the growth performance of this strain.

### 3.5. Identification of the Conversion Product of Arctigenin by the Oxygen-Tolerant Domesticated Strain

**Figure 6:**
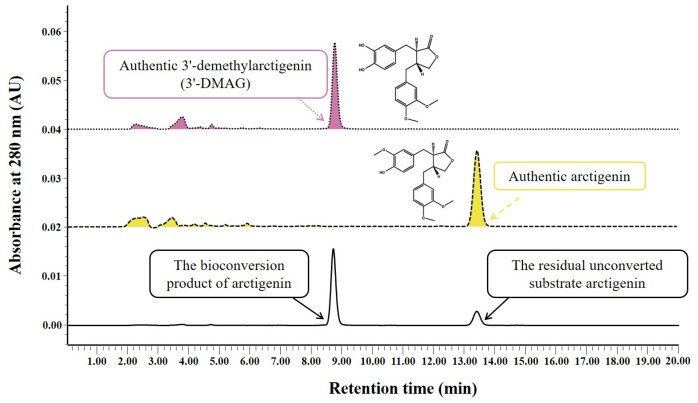
High-performance liquid chromatography (HPLC) elution profiles of arctigenin metabolism by the oxygen-tolerant strain Aeroto-AUH-df6 in the presence of atmospheric oxygen.

**Figure 7:**
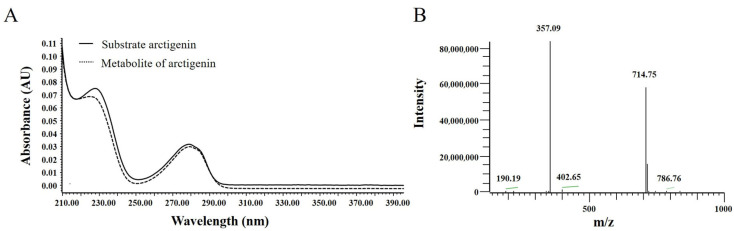
UV spectrum (**A**) and mass spectrum (**B**) of the arctigenin metabolite produced aerobically by the oxygen-tolerant strain Aeroto-AUH-df6.

After culturing the oxygen-tolerant strain Aeroto-AUH-df6 with the substrate arctigenin, the extract of the culture broth was analyzed by HPLC. The results showed that, in addition to the residual unconverted substrate arctigenin, a new characteristic chromatographic peak was detected at a retention time of 8.70 min (Figure 6). This chromatographic peak exhibited maximum UV absorption at 227 nm and 280 nm, which was completely consistent with the UV absorption characteristics of the demethylated product of arctigenin, 3′-DMAG (Figure 7A). Further mass spectrometry analysis of this new chromatographic peak was performed in negative ion mode (Figure 7B), yielding an ion peak signal of ESI^−^: *m*/*z* 357 ([M − H]^−^) corresponding to a molecular weight of 358. This exactly matches the theoretical molecular weight of the standard compound 3′-DMAG (molecular formula C_20_H_22_O_6_). In conclusion, based on the HPLC retention time, UV absorption spectrum, and mass spectrometry data, the metabolite with a retention time of 8.70 min was identified as 3′-DMAG.

To further clarify the chemical structure of this new compound peak, this study performed proton nuclear magnetic resonance (^1^H-NMR) and carbon-13 nuclear magnetic resonance (^13^C-NMR) characterization analyses on the purified target component. The detailed results are as follows:

^1^H-NMR (CDC_l3_, 400 MHz,): δ 2.46-2.64 (4H, m, H-2, 3, 7″), 2.85 (2H, d, *J* = 5.5 Hz, H-7′), 3.82–3.85 (6H, s, −OCH3 *2), 3.89 (1H, dd, *J* = 8.54, 7.6 Hz, H-4), 4.13 (1H, dd, *J* = 8.54, 7.8 Hz, H-4), 6.49 (1H, d, *J* = 1.94 Hz, H-2″), 6.52 (1H, dd, *J* = 8.19, 1.94 Hz, H-6′), 6.58 (1H, dd, *J* = 8.19, 1.94 Hz, H-6″), 6.67 (1H, d, *J* = 1.94 Hz, H-2′), 6.77 (1H, d, *J* = 8.19 Hz, H-5′), 6.78 (1H, d, *J* = 8.19 Hz, H-5″).

^13^C-NMR (CDC_l3_, 400 MHz): δ 179.9 (C-1), 149.0 (C-3″), 147.8 (C-4″), 144.1 (C-3′), 142.9 (C-4′), 130.1 (C-1′), 129.9 (C-1′), 121.7 (C-6′), 120.8 (C-6″), 116.2 (C-2′), 115.3 (C-5′), 111.9 (C-2″), 111.5 (C-5″), 71.9 (C-4), −Me 55.9, −Me 55.8, 46.6 (C-2), 41.0 (C-3), 38.3 (C-7″), 34.1 (C-7′).

Through combined interpretation of the ^1^H-NMR and ^13^C-NMR data, the target metabolite with a retention time of 8.70 min was precisely identified as 3′-DMAG. This result confirms that the oxygen-tolerant strain Aeroto-AUH-df6, obtained through oxygen-tolerance domestication process, can efficiently catalyze the conversion of the substrate arctigenin to 3′-DMAG under aerobic conditions in a conventional biochemical incubator.

### 3.6. Bioconversion Dynamics of the Oxygen-Tolerant Domesticated Strain

**Figure 8:**
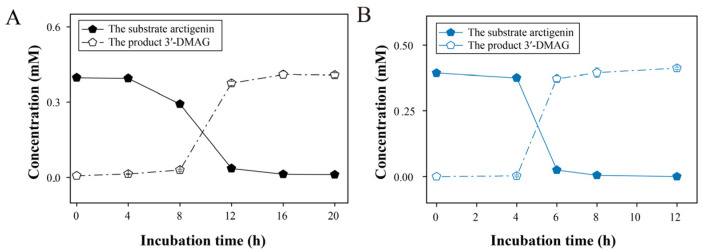
Bioconversion kinetics of the substrate arctigenin by the original strictly anaerobic strain AUH-JLD56 grown anaerobically (**A**) and by the oxygen-tolerant mutant strain Aeroto-AUH-df6 grown aerobically (**B**). The inoculum concentration was 10% (*v*/*v*). The initial concentration of arctigenin was 0.4 mM.

The strictly anaerobic bacterium *Blautia* sp. AUH-JLD56 and the oxygen-tolerant strain Aeroto-AUH-df6 were separately cultured with the substrate arctigenin in an anaerobic chamber and a conventional biochemical incubator, respectively. Samples were taken at different time points to dynamically monitor the substrate conversion process. The results showed that for the strictly anaerobic strain AUH-JLD56, the concentration of arctigenin decreased rapidly after 4 h of inoculation; during the period of 8~12 h post-inoculation, the concentration of the product 3′-DMAG increased rapidly in a linear manner, after which the rate of increase tended to level off (Figure 8A). In contrast, for the oxygen-tolerant strain Aeroto-AUH-df6, during the first 4~6 h post-inoculation, the substrate arctigenin was consumed rapidly in a linear fashion, while the product 3′-DMAG was simultaneously generated rapidly and linearly; thereafter, both the rate of substrate consumption and product synthesis slowed down significantly (Figure 8B).

### 3.7. Conversion Capacity of the Oxygen-Tolerant Strain Toward the Substrate Arctigenin

**Figure 9:**
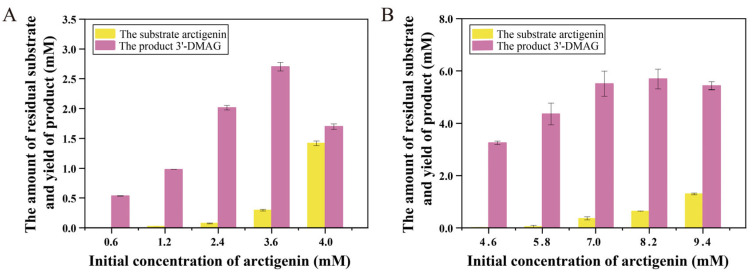
Bioconversion capacity of the original strictly anaerobic strain AUH-JLD56 grown anaerobically (**A**) and of the oxygen-tolerant mutant strain Aeroto-AUH-df6 grown aerobically (**B**). Compound 3′-DMAG is the metabolite of the substrate arctigenin.

To clarify the difference in conversion efficiency toward arctigenin before and after oxygen-tolerance domestication, the original strictly anaerobic strain *Blautia* sp. AUH-JLD56 and the oxygen-tolerant strain Aeroto-AUH-df6 were separately inoculated into pure BHI medium in an anaerobic chamber and a conventional biochemical incubator, respectively, and cultured with different concentrations of arctigenin. After 3 days of culture, the substrate conversion efficiency was determined by HPLC. The results showed that the conversion capacity of the non-domesticated wild-type strain AUH-JLD56 (Figure 9A) toward arctigenin was significantly inferior to that of the oxygen-tolerant strain Aeroto-AUH-df6 (Figure 9B). Under anaerobic conditions, the upper concentration limit for the efficient conversion of arctigenin by the wild-type strain was 3.6 mM, at which the average substrate conversion rate was 90.09% and the average product yield was 83.31%.

Under aerobic culture conditions, when the final concentration of arctigenin was ≤5.8 mM, the substrate was almost completely converted to 3′-DMAG. When the substrate concentration was increased to 7.0 mM, the average substrate conversion rate and average product formation rate of the oxygen-tolerant strain were 93.75% and 83.99%, respectively. At a concentration of 8.2 mM, the average conversion rate and formation rate were 90.01% and 77.15%, respectively. When the substrate concentration was further increased to 9.4 mM, the conversion capacity of the strain decreased significantly, with the average conversion rate dropping to 80.70% and the average product yield to 71.61%. In conclusion, under aerobic conditions, the upper concentration limit for the efficient conversion of arctigenin by the oxygen-tolerant strain Aeroto-AUH-df6 was 8.2 mM.

**Figure 10:**
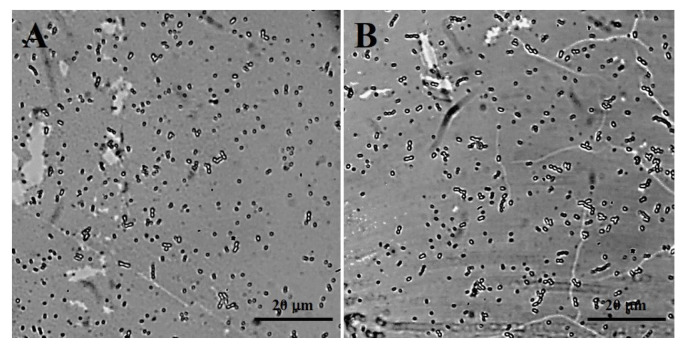
Light micrographs of the derived oxygen-tolerant strain Aeroto-AUH-df6 stained with Congo Red. Bacterial cells were sampled after 3 days of incubation in a conventional biochemical incubator under conditions without (**A**) or with (**B**) 8.2 mM arctigenin.

It can be seen from the above determination results of transformation capacity that with the continuous increase in the concentration of arctigenin (the substrate) added to the culture medium, both the substrate conversion rate and product yield of this strain showed a declining trend. Our research group previously obtained an oxygen-tolerant mutant strain *Clostridium* sp. Aeroto-AUH-JLC108 capable of the C-ring cleavage of soy isoflavone daidzein under aerobic conditions. Studies have confirmed that under high-concentration daidzein (2.4 mM) stress, this strain forms a protective film structure on the cell surface, mainly composed of polysaccharides [22]. This structure not only acts as a physical barrier against oxidative damage, but also slows down substrate permeation into the cells, thereby alleviating the toxic damage caused by high substrate concentrations. Based on this, we speculate that under high-concentration (e.g., 8.2 mM) arctigenin stress, the oxygen-tolerant mutant Aeroto-AUH-df6 may similarly form a protective film structure on its cell surface. To verify the above speculation, this study cultured the oxygen-tolerant mutant Aeroto-AUH-df6 in a conventional biochemical incubator, using cells without arctigenin addition as a control, and performed Congo Red staining analysis on cells exposed to a high concentration (8.2 mM) of arctigenin. The results showed that compared with cells without arctigenin (Figure 10A), the outer wall of cells treated with a high-concentration arctigenin did not form an obvious protective film structure (Figure 10B). However, the product yield showed a downward trend as the substrate concentration increased. Therefore, we speculate that under high-concentration arctigenin (8.2 mM) stress, this strain may form a light protective film structure on the cell surface. 

**Figure 11:**
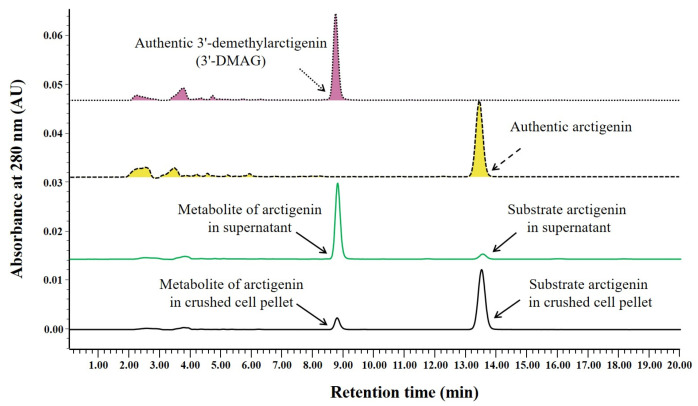
HPLC elution profiles of the substrate arctigenin and its metabolite 3′-DMAG extracted from the culture supernatant and from the crushed cell pellet.

To test this hypothesis, after the reaction, we centrifuged the culture medium, collected the cells for homogenization, and compared the substrate and product contents in the culture supernatant and the cell homogenate using HPLC. The HPLC detection results precisely confirmed the above hypothesis: the substrate peak was very large and the product peak was very small in the cell samples, which was completely opposite to the results in the supernatant (Figure 11).

### 3.8. Influence of Anaerobic Environment on Growth and Bioconversion Capacity of the Oxygen-Tolerant Strain

**Figure 12:**
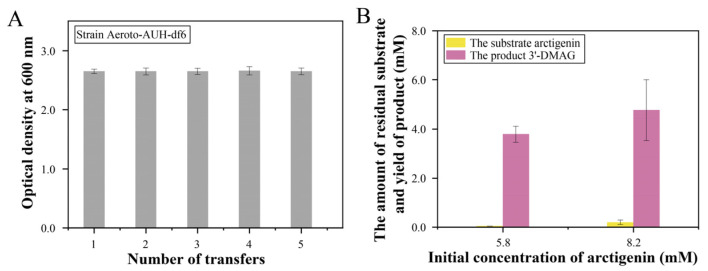
Influence of anaerobic conditions on growth (**A**) and bioconversion efficiency (**B**) of the oxygen-tolerant mutant strain Aeroto-AUH-df6.

The aerotolerant strain Aeroto-AUH-df6 was reactivated and consecutively subcultured five times under aerobic conditions. Once stable growth was established, the maximum OD_600nm_ and bioconversion capacity toward arctigenin were investigated. Under aerobic cultivation, the average maximum OD_600nm_ reached 2.43. When the initial arctigenin concentration in BHI medium was 5.8 mM, the average substrate conversion rate and product yield were 99.22% and 89.24%, respectively. Increasing the substrate concentration to 8.2 mM reduced these values to 91.12% and 77.91%, respectively. To evaluate the influence of anaerobic conditions on growth and bioconversion, the aerobically adapted strain was serially transferred five times in an anaerobic chamber, with OD_600nm_ measured after each passage. Biomass remained stable across all transfers, with a consistent average maximum OD_600nm_ of 2.45. Following anaerobic acclimation, the strain was inoculated into fresh BHI medium containing different arctigenin concentrations to assess substrate conversion under anaerobic conditions. At an initial concentration of 5.8 mM, the average conversion rate and product yield were 98.92% and 66.00%, respectively; at 8.2 mM, the corresponding values were 96.04% and 60.61%, respectively (Figure 12).

These findings indicate that while Aeroto-AUH-df6 achieves comparable biomass under both aerobic and anaerobic conditions, its bioconversion efficiency differs markedly—most notably, a substantial reduction in product yield under anaerobiosis. An additional observation revealed that 7 h after the first transfer to anaerobic conditions, the aerobic group exhibited a 1.73-fold higher biomass than the anaerobic group, confirming a significantly slower growth rate in the absence of oxygen. It is therefore hypothesized that the aerotolerance-acclimated strain has adapted to aerobic growth; its abrupt shift to anaerobic conditions imposes environmental stress, not only decelerating growth but also severely impairing the production efficiency of the metabolite 3′-DMAG. Future studies will examine whether prolonged anaerobic subculturing allows the strain to gradually adapt and improve its bioconversion efficiency toward arctigenin.

## 4. Discussion

*Arctium lappa* L. fruit is a traditional Chinese medicinal herb with complex chemical constituents, among which the lignan compound arctiin is its core characteristic active ingredient. Since traditional Chinese medicines are mostly administered orally, arctiin enters the body and is preferentially degraded by the gut microbiota in the intestine, generating various derivatives. In 2001, Heinonen et al. [25] first demonstrated that human fecal microbiota possess the ability to metabolize arctiin, converting it into enterolactone and other active metabolites. In 2013, our research group isolated a strictly anaerobic bacterium, *Blautia* sp. AUH-JLD56, from a human fecal sample. This strain can sequentially deglycosylate arctiin under anaerobic conditions to produce arctigenin, and further specifically convert arctigenin into 3′-DMAG. Our in vitro antioxidant experiments showed that in the concentration range of 0.025~0.100 mM, 3′-DMAG exhibited significantly stronger DPPH radical scavenging activity than its precursor arctigenin (*p* < 0.01) [13]. Subsequent studies also revealed that 3′-DMAG had a significantly stronger inhibitory effect on human hepatocellular carcinoma HepG2 cells in vitro than arctigenin. Furthermore, using a mouse xenograft model of liver cancer, we found that 3′-DMAG significantly suppressed tumor growth without obvious toxic side effects [6]. The metabolite 3′-DMAG of arctigenin possesses excellent bioactivity, but its large-scale preparation by chemical synthesis is currently not feasible. The isolation of strain *Blautia* sp. AUH-JLD56 offers a possible approach for the microbial transformation synthesis of 3′-DMAG. However, this wild-type strain is strictly anaerobic and highly sensitive to oxygen; both its growth and substrate conversion require strict anaerobic conditions. This oxygen sensitivity greatly limits its industrial application.

To address the above problem, this study used the strictly anaerobic bacterium *Blautia* sp. AUH-JLD56 as the starting strain, conducted long-term oxygen tolerance acclimation, and successfully obtained an oxygen-tolerant mutant strain, named *Blautia* sp. Aeroto-AUH-df6. Compared with the wild-type strain AUH-JLD56, this oxygen-tolerant mutant exhibited significant changes in cell morphology, physiological and biochemical characteristics, substrate conversion rate, and maximum conversion capacity. Under anaerobic conditions, the wild-type strain AUH-JLD56 efficiently converted arctigenin, with a maximum conversion concentration of 3.6 mM, an average conversion rate of 90.09%, and a product yield of 83.31%. In contrast, the oxygen-tolerant mutant Aeroto-AUH-df6 efficiently converted arctigenin under aerobic conditions, achieving a maximum conversion concentration of 8.2 mM, with an average conversion rate of 90.01% and a product yield of 77.15%. Notably, when the final concentration of arctigenin was ≤7.0 mM, the product yield of the oxygen-tolerant mutant was essentially consistent with that of the wild-type strain under anaerobic conditions. However, when the substrate concentration was further increased to 9.4 mM, the product yield further decreased to 71.61%.

Our previous studies have confirmed that 5 mM arctigenin and its metabolite 3′-DMAG exhibit no antibacterial activity against *Staphylococcus aureus* (ATCC27217), *Salmonella paratyphi* (CMCC50001), or *Escherichia coli* (CICC10372) [13]. In the present study, when the final concentration of arctigenin was 7.0 mM, the strain achieved an average substrate conversion rate of 93.75% and an average product yield of 83.99%, which was essentially consistent with the conversion level of the wild-type strain under anaerobic conditions, further indicating that 7.0 mM arctigenin and its conversion product 3′-DMAG did not cause obvious toxic damage to Aeroto-AUH-df6. Based on the changes in product yield, when the substrate concentration was increased to 8.2 mM and 9.4 mM, the product yield showed a significant decreasing trend, suggesting that the substrate concentration at 8.2 mM already exerted some toxicity on the cells. Although our Congo Red staining results indicated that no obvious protective film was formed when cells of the aerotolerant mutant strain Aeroto-AUH-df6 were exposed to a high concentration of arctigenin (Figure 10), a certain amount of untransformed substrate arctigenin was detected in the harvested and ground cells of Aeroto-AUH-df6 via HPLC analysis (Figure 11). These findings verify that 8.2 mM arctigenin exerts moderate cytotoxicity against Aeroto-AUH-df6 cells, prompting the strain to synthesize protective membrane structures. However, because the toxic stress intensity was limited, the protective film structure formed by the cells was not prominent. From the perspective of compound structure, the product has one more hydroxyl group than the substrate, suggesting that the water solubility of the product may be higher than that of the substrate. If so, the amounts of unconverted substrate and unexported product retained in the protective film may not be equal; instead, the substrate with lower water solubility would account for a higher proportion. Finally, we measured the maximum water solubility of the substrate and product at 37 °C, and the results were as expected: the solubility of the substrate (arctigenin) was 0.153 g/L, and the maximum solubility of the product was 0.870 g/L (not yet reported). In short, the higher water solubility of the product allows it to more easily “escape” from the protective film, while the substrate is more likely to be “trapped” inside the protective film.

Urease catalyzes the hydrolysis of urea to ammonia and is a pivotal enzyme in the biological nitrogen cycle. The influence of urease activity on bacterial pathogenicity is not an intrinsic property of bacterial strains: in opportunistic pathogens, urease generally augments virulence potential, while in non-pathogenic commensals, it provides physiological benefits. In *Helicobacter pylori*, which thrives in the highly acidic gastric environment, urease functions both as an essential survival factor and a major virulence determinant. Tsuda et al. [26] constructed a urease-null mutant of *H. pylori*, and through nude mouse inoculation experiments, directly verified that urease is absolutely required for gastric colonization. Similarly, De Koning-Ward and Robins-Browne [27] deleted the urease gene cluster in *Yersinia enterocolitica* and observed that the mutant exhibited 1000-fold lower acid tolerance in vitro compared to the wild-type strain, indicating that urease promotes the invasive virulence of this pathogen by enhancing its survival in gastric acid. However, when Ryvchin et al. [28] analyzed the distribution of urease-producing bacteria in patients with inflammatory bowel disease (IBD) via fecal metagenomics, they found no significant difference in overall gut abundance between IBD patients and healthy controls. In healthy individuals and most IBD patients, the urease-producing microbiota are dominated by acetogenic genera such as *Blautia* and *Ruminococcus*. In contrast, IBD patients tend to show a shift in these microbial populations toward Bacilli and Proteobacteria, or even a complete loss of such communities. In 2025, Firth and colleagues [29] further demonstrated that *Blautia* strains harboring urease-encoding genes can directly assimilate carbon skeletons derived from urea to synthesize SCFAs. In contrast, *Klebsiella pneumoniae* strain MH258 and *Proteus mirabilis* strain MH42F also possess the activity of hydrolyzing urea, but they lack the acetogenic pathway and thus cannot produce SCFAs from carbon sources derived from urea. Therefore, urease-positive *Blautia* exhibit a distinct metabolic strategy for urea utilization, which fundamentally sets them apart from urea-degrading pathogenic bacteria.

Our findings reveal that the original strain *Blautia* sp. AUH-JLD56 is urease negative, whereas the aerotolerant mutant Aeroto-AUH-df6 acquires urease activity and exhibits a urease positive phenotype. Under anaerobic conditions, the wild-type strain AUH-JLD56 shows only weak acidification of the medium, with the pH decreasing from 7.0 to 6.5 after incubation. In contrast, the aerotolerant mutant displays a stronger acid-producing capacity under aerobic cultivation, lowering the pH from 7.0 to 5.1 (Figure 2), with acetic acid as its primary metabolic product (Figure 3). Dynamic pH monitoring reveals a staged pH variation pattern during mutant cultivation: the pH drops continuously within the first 2 h after inoculation, rises slightly between 2 and 4 h, then decreases steadily again, and finally stabilizes at approximately 5.1 (Figure 2B). Based on these phenotypic characteristics, we hypothesize that the newly acquired urease activity in Aeroto-AUH-df6 generates ammonia through urea hydrolysis, which neutralizes local acidity, thereby alleviating acid stress, enhancing environmental adaptability, and improving the survival competence of the strain.

On the other hand, acid production by the aerotolerant mutant Aeroto-AUH-df6 may aid its adaptation to aerobic conditions. Although acetic acid itself has no direct antioxidant activity, it can indirectly regulate intracellular redox homeostasis. As early as 1971, O’Brien and Morris [30] first demonstrated that the key strategy of *Clostridium acetobutylicum* in achieving short-term oxygen adaptation is the continuous synthesis of acetic acid: acetate helps maintain the intracellular reducing power balance and eliminates oxygen toxicity, thereby ensuring short-term survival of the bacterium under aerobic stress. In 2002, Karnholz et al. [31] further found that some strictly anaerobic acetogenic bacteria can synthesize acetate via the acetyl-CoA pathway to adapt to low-oxygen environments. In 2007, Briukhanov and Netrusov [32] systematically reviewed the stress effects of aerobic environments on strictly anaerobic bacteria and clearly identified acetogenesis as the main metabolic strategy for short-term oxygen tolerance adaptation in obligate anaerobes, a process that achieves adaptive regulation to aerobic stress by maintaining redox balance and coupling with reactive oxygen species scavenging mechanisms. In the present study, the oxygen-tolerant mutant Aeroto-AUH-df6 actively redirected its carbon metabolic flux, prioritizing the use of carbon sources for acetate synthesis to alleviate intracellular oxidative stress. This “metabolic trade-off” mechanism well explains why the mutant exhibited a significantly broadened carbon source utilization spectrum while its biomass did not increase but rather decreased: carbon sources and energy were largely used for acetate production to combat oxygen toxicity, rather than for growth and reproduction. How this mutant regulates acetate synthesis under aerobic conditions remains to be further investigated.

## 5. Conclusions

Through long-term oxygen tolerance domestication of the strictly anaerobic wild-type *Blautia* sp. AUH-JLD56, an oxygen-tolerant mutant strain was successfully obtained. Phenotypic analysis showed that the biomass of the mutant under aerobic conditions was slightly lower than that of the wild-type under strict anaerobic conditions. Compared with the wild-type, the mutant exhibited an accelerated aerobic growth rate and enabled the stable conversion of arctigenin. Moreover, the maximum concentration of arctigenin converted by the mutant under aerobic conditions (8.2 mM; average conversion rate and product yield: 90.01% and 77.15%, respectively) was significantly higher than that achieved by the wild-type (3.6 mM, average conversion rate and product yield: 90.09% and 83.31%, respectively). The oxygen tolerance domestication method developed in this study achieves aerobic adaptive modification while preserving the demethylation conversion function of the strain, which is of great value for promoting the aerobic development and industrial application of gut strict anaerobes with specific bioconversion functions.

## Figures and Tables

**Table 1:** Oxygen-tolerant domestication procedures for bacterial strain *Blautia* sp. JLD56.

Different Stages	Ascorbic Acid (%, *m*/*v*)	L-Cysteine (%, *m*/*v*)	Agar (%, *m*/*v*)	Number of Transfers
Initial stage	0.15	0.15	0.08	5
Ascorbic acid reduction stage	0.14	0.15	0.08	5
	0	0.15	0.08	70
L-cysteine reduction stage	0	0.14	0.08	5
		0	0.08	70
Agar reduction stage	0	0	0.07	5
			0	35
End stage	0	0	0	5

**Table 2:** Physiological and biochemical indicators of the original strictly anaerobic wild-type strain *Blautia* sp. AUH-JLD56 and the oxygen-tolerant mutant strain Aeroto-AUH-df6.

Identification	Strain AUH-JLD56	Strain Aeroto-AUH-df6	Identification	Strain AUH-JLD56	Strain Aeroto-AUH-df6
Urease	−	+	Arabinose	−	+
Amylohydrolysis	−	−	Mannose	−	+
Indole production	−	−	Trehalose	−	+
H_2_S production	−	−	Melezitose	−	+
Glucose	−	−	Cellobiose	−	+
Maltose	−	−	Gelatin	−	−
Sucrose	−	+	Esculin	−	−
Xylose	−	+	Glycerol	−	+
Raffinose	−	+	Salicin	−	+
Rhamnose	−	+	Sorbitol	−	+

Note: The symbol “+” indicates positive, while “−” indicates negative results.

## Data Availability

The original contributions presented in the study are included in the article, and further inquiries can be directed to the corresponding authors.

## Abbreviations

The following abbreviations are used in this manuscript:

3′-DMAG	3′-Demethylarctigenin
HPLC	High-performance liquid chromatography
